# Physicochemical considerations for bottom-up synthetic biology

**DOI:** 10.1042/ETLS20190017

**Published:** 2019-08-28

**Authors:** Wojciech Mikołaj Śmigiel, Pauline Lefrançois, Bert Poolman

**Affiliations:** Department of Biochemistry, University of Groningen, Groningen Biomolecular Sciences and Biotechnology Institute, Nijenborgh 4, 9747 AG Groningen, the Netherlands

**Keywords:** bottom-up synthetic cells, macromolecular crowding, membrane reconstitution, physicochemical homeostasis, synthetic biochemistry, vesicles

## Abstract

The bottom-up construction of synthetic cells from molecular components is arguably one of the most challenging areas of research in the life sciences. We review the impact of confining biological systems in synthetic vesicles. Complex cell-like systems require control of the internal pH, ionic strength, (macro)molecular crowding, redox state and metabolic energy conservation. These physicochemical parameters influence protein activity and need to be maintained within limits to ensure the system remains in steady-state. We present the physicochemical considerations for building synthetic cells with dimensions ranging from the smallest prokaryotes to eukaryotic cells.

## Introduction

Cells are products of billions of years of evolution. The molecular machinery of each cell is a complicated network, optimized for the particular set of conditions the organism faces. Spatiotemporal interactions between DNA, RNA, proteins, metabolites, ions and other molecules result in emergent properties necessary for life. The concentration of all these molecules in the confined space of a cell is very high, which, through intermolecular interactions, influences the physicochemical properties of the cytoplasm. To enable the proper working of a cell the pH, ionic strength, macromolecular crowding and metabolic energy need to be kept within narrow windows, which we refer to as physicochemical homeostasis.

The complexity of cells makes it difficult to study the underlying molecular mechanisms *in vivo.* In synthetic biology, there are two ways to reduce cell complexity, that is, by removing genes in living cells (top-down) or by constructing life-like systems from molecular components (bottom-up) [1–3]. By reconstructing living cells from the ground up we can learn a lot, not only about the components and their interactions, but also how cells evolve and how we can better utilize them for technological purposes. A challenge for the bottom-up synthetic biology is to construct life-like systems capable of maintaining physicochemical homeostasis with a minimal number of components. Here, we review the challenges that one faces in realizing physicochemical homeostasis within bottom-up constructed synthetic cells.

## Physicochemical nature of the cell lumen

### Water

Biochemical reactions depend on water. The network of hydrogen bonds forces water to arrange around larger molecules, which drives the macromolecular hydrodynamic volume minimization. The hydrophobic effect, created by repulsive interactions between water and non-polar molecules, stabilizes biological membranes and drives the folding of water-soluble proteins [4]. Additionally, high concentrations of some sugars and quaternary ammonium compounds can act as co-solvents and contribute to the stability of biomolecular systems [5,6].

### Internal pH

pH can affect the structure, function and reactivity of macromolecules. In cells, a low internal pH causes protein and DNA damage [7]. The solubility of proteins drops when the isoelectric point (pI) is close to the internal pH [8]. Furthermore, the catalytic activity of many enzymes and membrane transporters is highly dependent of pH. Most cells maintain their internal pH in the range of 7–7.5, but extremophiles can handle more acidic or alkaline internal environments [9]. Amino acids and phosphates buffer the internal pH [9], yet all cells require active proton pumps and ion exchange mechanisms for pH homeostasis [10]. Regulation of the internal pH is intertwined with the control of electrochemical proton gradient (Δp) as the pH gradient (ΔpH) is a component of Δp, and thus any charge movement (not just protons) across the membrane will directly or indirectly affect the internal pH (Δp = ΔΨ − ZΔpH, where ΔΨ is the membrane potential and *Z* equals 58 mV at 298 K). Redox, light energy [11–13] or ATP can be used to pump protons out [9,14], but it is also possible to change the cytoplasmic pH through decarboxylation and deamination reactions [9]. A large part of the acidification of the cytoplasm originates from the uptake of protons via solute-proton symporters (e.g. importing nutrients) or antiporters (exporting waste products) [14]. Key components of pH homeostasis are Na^+^/H^+^ antiporters [9,10,14] or equivalent systems that sense and respond to changes in the internal pH.

### Ionic strength

The ionic strength (I) of a solution impacts the properties of macromolecules by ionic screening, which affects the strength and distance of electrostatic and hydrophobic interactions [15,16]. The ionic strength of the cytoplasm of *Escherichia coli* is 0.2–0.3 M, but in Gram-positive bacteria like *Bacillus subtilis* and *Lactococcus lactis* it is higher, because the salt concentrations (e.g. K^+^) are higher [17], which also contributes to a higher turgor pressure [15]. The type of ions and their concentrations influence the solubility of macromolecules, which is known as the Hofmeister effect [18,19]. The Hofmeister series is a classification of ions in order of their ability to decrease (salt-out) or increase (salt-in) protein solubility. The most abundant cations present in cells are potassium, magnesium and sodium ions [20]. The most abundant small inorganic anions are phosphate and chloride ions [20], in many cells glutamate and organic phosphates also contribute significantly to the negative charge (and buffering capacity) [20]. Net ion transport*,* e.g. in the form of potassium uptake or release, influences the internal ionic strength but also ΔΨ and thus the pH gradient across the membrane. Hence, pH and ionic strength homeostasis are strongly connected.

### Excluded volume and macromolecular crowding

**Figure 1.**
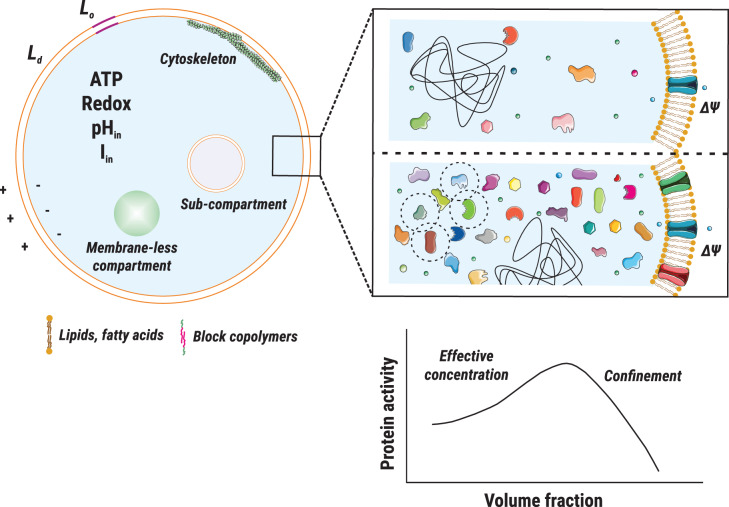
Schematic representation of physicochemical effects at different scales. The main physicochemical parameters and compartmentalization of the vesicle lumen are indicated on the left; two scenarios of crowding are indicated on the right, with a non-crowded situation on the top and a crowded case on the bottom. Schematic of the influence of macromolecular crowding on protein activity, adapted from [22], is indicated in the bottom panel.

In cells, each protein is affected by the presence of other macromolecules. The volume inaccessible to a protein is called excluded volume and depends on the size of the protein of interest and on the macromolecular volume fraction [21]. From the perspective of a particular protein, an increase in excluded volume increases its effective concentration, while at the same time decreasing its lateral diffusion coefficient [22]. Excluded volume can affect the folding, the oligomeric state and the reaction rates of enzymes [23,24]. An increase in excluded volume (e.g. upon osmotic upshift) can confine macromolecules to small volumes bordered by tightly packed molecules [25]. For most reactions, a rise in excluded volume increases the activity until a point where the reaction becomes diffusion limited (Figure 1). It has been shown that increasing concentrations of dextran resulted in higher activity of RNA polymerase in solution and in synthetic cells, but at some critical value the enzyme diffusion became limiting [26]. Similarly, a fill-and-shrink sequence enhances protein synthesis in vesicles, when crowding is increased by osmotic upshift [27]. In the *E. coli* cytoplasm, the concentration of macromolecules is at ∼250 g L^−1^, taking up ∼16% of the total internal volume [28,29]. The average distance between the surfaces of proteins in such a cell is in the range of 1–2 nm, but becomes smaller when cells are exposed to osmotic upshift stress. Under these conditions the distances between macromolecules are in the range where hydration forces and electrostatic interactions are stronger [30]. Hence, controlling ionic strength and pH in connection with macromolecular crowing is necessary to minimize protein aggregation and salting-out effects. In current synthetic systems, the concentration of macromolecules is usually much lower than in cells resulting in distances between molecules in the order of hundreds of nanometers. Under such conditions, excluded volume effects are insignificant [31]. With increasing complexity of bottom-up constructed synthetic cells, the solutions will become more crowded; excluded volume and other crowding effects become relevant. Cells control the concentration of cytoplasmic macromolecules by, for instance, controlling cell volume through import or excretion of osmolytes, but an overarching mechanism of crowding homeostasis is yet to be discovered [32]. Upon osmotic upshift the volume of cells decrease, which is countered by importing potassium ions and/or by taking up and/or synthesizing compatible solutes [5,6]. Compatible solutes increase the internal osmotic pressure, causing water to flow back in, allowing cells to regain their volume. They also positively impact the stability of proteins and other macromolecular complexes, and at high concentrations they act as co-solvents rather than small molecule effectors [33]. A study in individual cells has shown that the diffusion coefficients of cytoplasmic proteins follow a unimodal distribution, indicating that cells strive to maintain crowding at a certain level [17,29]. The mobility of a single probe in three prokaryotes led to a similar conclusion but also showed that macromolecular crowding and ionic strength are linked [34].

### Metabolic energy conservation

Physicochemical homeostasis, like any cellular homeostatic process, consumes a considerable amount of ATP and/or metabolic energy conserved in the form of the electrochemical ion potential (Δ*p* and/or Δ*s* = ΔΨ − ZΔpNa) across the membrane [9]. Metabolic energy generation in living cells is complex and requires numerous enzymes and cofactors [35]. Nature offers alternative mechanisms to conserve metabolic energy through simple metabolic conversions (deamination of amino acids, oxidation of carboxylic acids) or the use of light, which has been recently reviewed [36]. Sustainable synthesis of ATP from the breakdown of arginine in a vesicle system and controlled import and export of reaction products has been demonstrated [37] (see Box I), yet more ways of generating metabolic energy should be explored, e.g. by combining simple, independent pathways for synthesis of ATP and generation of an electrochemical ion gradient.

Box 1. **Long-term metabolic energy conservation in vesicles**

Any living cell maintains the pH, ionic strength, osmotic pressure, macromolecular crowding and phosphorylation potential within limits to allow the enzymes and other components to function near their optimum. Open systems need continuous feed, drain and energy dissipation to allow metabolic reactions to remain away from equilibrium. The arginine breakdown pathway has been co-reconstituted with the ionic strength-gated ATP-driven osmolyte transporter OpuA to allow for vesicle expansion and restoration of the physicochemical conditions upon exposure to osmotic stress. When the vesicles are exposed to an increasing medium osmolality, they shrink, resulting in an increase in concentration of internal components. Under these conditions the pathway functions sub-optimally and the enzymes are gradually inactivated. However, when the ionic strength reaches a critical value, OpuA is activated and glycine betaine is pumped inside, which is accompanied by passive influx of water into the vesicles. This increases the volume, reduces the ionic strength and stabilizes the internal pH. The system can supply synthetic cells with ATP up to at least one day [37] (Figure 4).

Lee et al. have developed so-called artificial organelles to maintain and control the electrochemical proton gradient and to synthesize ATP [11]. They used the F_0_F_1_-ATP synthase with two photoconverters, a photosystem II and proteorhodopsin. The three proteins were reconstituted in small lipid vesicles (artificial organelles) with the F_1_ domain of the ATP synthase on the outside. Upon activation of photosystem II by red light, protons are generated inside the vesicles (the interior becomes positive and acidic), and the proton motive force drives the synthesis of ATP. Activation of proteorhodopsin by green light dissipates the proton motive force, which impedes the synthesis of ATP. The artificial organelles were encapsulated in giant vesicles to provide them with ATP and drive endergonic reactions. Similarly, a simpler system was developed by Berhanu et al., where bacteriorhodopsin was used for proton translocation. ATP generated by the F_0_F_1_-ATP synthase was later used as a fuel for protein synthesis [12].

## Physicochemical properties of membranes

### Cell and vesicle boundary

The inside of a cell is separated from the outside by a semipermeable bilayer, the lipid membrane, and here a number of physicochemical conditions also applies. The amphiphilic nature of the lipids enables spontaneous formation of the bilayer structure in aqueous media. A typical cell membrane is composed of hundreds of different types of lipids (in mammalian cells, ∼60% glycerophospholipids, ∼10% sphingolipids, 0.1–40% sterols) [20], which determine the curvature, thickness, rigidity and fluidity of the membrane. In general, the longer and more saturated the hydrophobic chains are, the less permeable a membrane is for solutes [38]. In cells, a certain degree of unsaturation is required to maintain sufficient fluidity and enable the embedded proteins to be functional [39]. Each lipid has a specific transition temperature (*T*_m_). Two main phases (crystalline-ordered *L_o_* and liquid-disordered *L_d_*) define the degree of fluidity and order of the membrane, the thickness and to some extent, its permeability [40,41]. Indeed, when the membrane fluidifies, lipids can accommodate their shape, enabling the passage of solutes, therefore, increasing the membrane permeability for some small solutes [42,43].

### Membrane proteins

Membrane proteins are essential for signal information from the environment, import nutrients, export waste and toxic products. They maintain and utilize the electrochemical ion gradients and ATP (or phosphorylation potential) as a driving force. From *in vitro* studies, it appears that particular lipid headgroups (e.g. PG or PS) or intrinsic curvature (e.g. PE) are crucial for the activity of membrane transporters [44]. Similarly, the membrane protein can have a dependence on acyl chain length and/or degree of (un)saturation, and these are therefore essential conditions to be considered in the design of a synthetic cell [45,46].

### Membrane domains

Depending on the transition temperature of the domain-forming lipid, different phases can coexist in the membrane (when at least two different lipids are present in the membrane). Lipid (nano)domains are dynamic regions of ‘different chemical compositions and/or physical properties compared with their surrounding environment’. They are formed and maintained by many factors (e.g. electrostatic interactions, embedded membrane proteins, curvature…, for a recent review, see [47]). This directly impacts the fluidity and the thickness of the membrane locally [41]. Consequently, the passive permeability of solutes and the activity of embedded proteins may be different inside and outside of the (nano)domains [48]. In eukaryotic cells, the lipid domains play a role in protein trafficking, cell signaling and interactions with pathogens [47]. Another factor to consider when building a synthetic cell is asymmetry of lipids over the two membrane leaflets. In mammalian cells, anionic lipids like PS and PIPs and the non-bilayer lipid PE are more abundant in the inner leaflet, whereas PC and sphingolipids are most abundant in the outer leaflet [44,49]. Interestingly, interaction of actin filaments with transmembrane proteins affects the inner and outer leaflet composition. Transmembrane proteins are arrayed into diffusion barriers (‘pickets’), influencing the mobility of membrane proteins and outer leaflet lipids [47,50–53].

## Physicochemical considerations for synthetic cells

### Reproducing cellular compartmentalization

**Figure 2.**
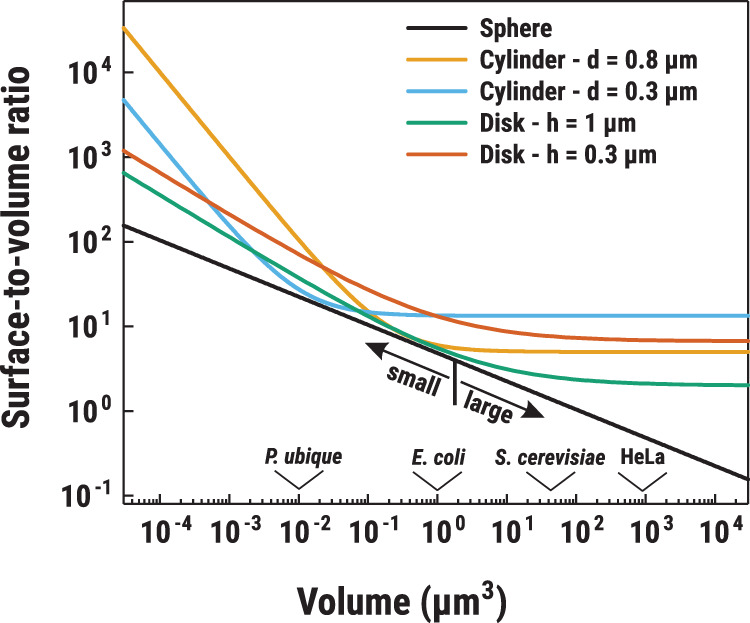
Discoidal and filamentous structures offer a better scaling of surface area with volume than spheres. Graph showing the dependence of the surface-to-volume ratio on volume of spherical (black line) and cylindrical (yellow, cyan, green and orange lines) compartments. Arrows on the black line indicate the arbitrary division of small (<2 µm diameter) and big (>2 µm diameter) vesicles. Base diameters of the cylinders were fixed at 0.8 µm (yellow) and 0.3 µm (cyan) to represent cell-size filaments and lipid nanotubes, respectively. For green and orange lines, cylinder heights were fixed at 1 µm and 0.3 µm, respectively, to represent discoidal cells. Arrows on the *x*-axis indicate volumes of several organisms: *P. ubique* (0.01 µm^3^, [63]), *E. coli* (1.1 µm^3^; BNID:100004), haploid *Saccharomyces cerevisiae* (37 µm^3^; BNID:100430) and HeLa (940 µm^3^; BNID:106664) cells. The surface-to-volume ratio for a sphere is given by the equation: *A*/*V* = 3/(3*V*/4π)^1/3^; for a cylinder with a fixed base diameter the equation is as follows: *A*/*V* = (4/*d* + 2/πd^2^)/*V*; for a cylinder with a fixed height: *A*/*V* = (2π(*V*/*h*π)^1/2^*h* + *V*/2. *V* is the volume, *d* is the cylinder base diameter and *h* is the cylinder height.

Multiple systems have been developed to mimic cell compartmentalization; they are commonly referred to as ‘vesicles’ due to their (usually) spherical shape. Different types of vesicles can be made, based on diverse amphiphilic molecules (liposomes, polymersomes, for a recent review in a synthetic cell perspective, see [54]), or droplet-based structures (coacervates [27,55–58], layer-by-layer capsules [59–61]). Liposomes are the closest model of native cellular membranes, and they have been used for studying membrane transport, membrane fusion, cell adherence, membrane trafficking and molecular recognition [62]. A wide range of vesicle types and sizes are in use for the construction of synthetic cells. Two main size ranges of vesicles are considered here. We refer to small vesicles when diameters are <2 µm (e.g. small- and large-unilamellar vesicles, SUVs and LUVs), and to large vesicles for structures with diameters >2 µm (e.g. giant-unilamellar vesicles, GUVs). We can compare the size of these vesicles with living cells. One of the smallest bacteria, *Pelagibacter ubique,* has a volume of ∼0.01 µm^3^ [63], which would correspond to a 0.26 µm diameter if it was a spherical cell; while *E. coli* with a volume of 1 µm^3^ (Bionumbers ID BNID: 100004) would give a diameter of 1.24 µm; other examples are given in Figure 2.

### Surface-to-volume ratio and membrane permeability

The choice of the vesicle size is a key parameter for the construction of a synthetic cell, since each size comes with different constraints. High-throughput analytical studies are typically conducted with small vesicles since they are easily formed in large amounts and mechanically robust. Recent microfluidic developments enable high-throughput studies on large vesicles [64]. Large vesicles can be monitored with light microscopy but their low surface-to-volume ratio can pose a problem for sufficient molecule transport (Figure 2). In this review, we omit vesicle fusion (exo- and endocytosis) as means to transport cargo across membranes, for reviews, see [3,65,66]. For certain molecules cells do not require transporters, as they diffuse passively across the membrane (e.g. water, O_2_, N_2_, urea, glycerol, weak acids and bases), while salts, polar solutes and macromolecules require transport mechanisms. For instance, the permeability coefficient of a typical lipid membrane for water is ∼10^−2^ cm s^−1^, whereas for K^+^ and Cl^−^, the values are ∼10^−12^ and ∼10^−9^ cm s^−1^, respectively [67]. Weak acids and bases represent intermediate cases with permeability coefficients in the range of 10^−5^ to 10^−3^ cm s^−1^. Even without a specific transport mechanism a gradient of weak acids (and bases) is formed when a pH gradient is present across the membrane [68,69]. It has been shown that membranes prepared from long-chain fatty acids are much more permeable to solutes than those of phospholipids, and even sugars permeate such membranes at significant rates [70]. Highly permeable membranes prevent electrochemical ion and solute gradients to be maintained. On the other hand, permeable vesicles enable passive influx of solutes, without the need for specific transporters.

### Choice of amphiphiles

The choice of the lipid composition is crucial for constructing synthetic cells, especially so when integral membrane proteins are incorporated into the membrane. In living cells, the lipid composition is complex and dynamic [41]. The cell membrane is tuned to the environmental conditions and provides an adaptable scaffold for membrane proteins that need to be reproduced in synthetic cells. Polymersomes or coacervates provide different physicochemical parameters than lipid membranes, yet some membrane proteins have been successfully reconstituted in polymersomes [71]. Isoprenoid chains, mostly found in archaeal cells, could provide higher robustness to the membranes of synthetic cells [72]. Lipid- and fatty acid-based vesicles are chemically closer to bacterial and eukaryotic membranes than those prepared from non-biological amphiphiles, but they are less stable than membranes formed from e.g. block-copolymers [54]. Mixtures of different types of amphiphiles may offer a compromise between high activity and high stability, but functional studies of membrane transport remain to be done. Furthermore, if the membrane is to grow, all the necessary membrane components have to be synthesizable with the use of enzymes or fed into the system with subsequent insertion into the membrane. Phospholipid biosynthesis in liposomes has been demonstrated to work with purified enzymes [73] and enzymes synthesized from an encapsulated cell-free transcription-translation system [74]. So far, the amount of phospholipids synthesized are low, the synthesis and insertion of membrane proteins remain a challenge [75,76].

### Vesicle stability

Liposomes are known to be intrinsically metastable, causing membrane rupture and/or fusion over time (hours to weeks for liposomes) [54], which can result in loss of content. Thus, synthesis and transport of the cell constituents have to be sufficiently fast to meet the demands for growth and nonspecific leakage. Temperature, osmotic stress, electric field [77], light exposure [78] and pH changes [79] are parameters that can oxidize lipids and/or destabilize the membrane [80]. Hybrid systems made of lipids and synthetic block-copolymers may be a good alternative to overcome some of these limitations [56,71,81].

### Sub-compartmentalization

Intracellular compartments are one of the ways cells deal with separating processes that would otherwise interfere with one another. Many sub-compartmentalized systems have been developed for use in synthetic cells. Some are based on the encapsulation of small vesicles within larger ones (e.g. liposomes-in-liposomes, polymersomes-in-polymersomes) [82]. Others consist of membrane-less compartments, formed by phase separation (e.g. coacervates). This mechanism is analogous to the phase separation of soluble proteins in living cells [83]. Nonspecific release of contents between inside and outside of sub-compartments can be activated, for instance, by light, change of pH, osmotic pressure or temperature [55,84,85].

### Size and shape of vesicles

**Figure 3.**
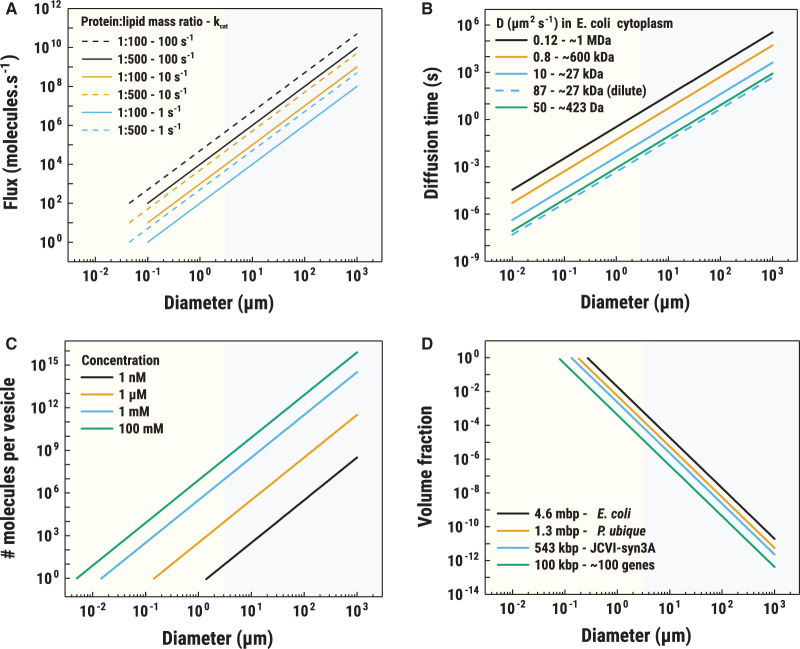
Impacts of vesicle size on some physicochemical parameters. (A) High protein-to-lipid ratios allow for smaller synthetic cells. The scaling of the flux of molecules imported into vesicles is shown. Flux is given by the equation: *J *= (M*_lip_*π*d*^2^*r_pl_k_cat_*)/*M_prot_A_lip_*; where *M_lip_* is the molecular mass of the lipid, *d* is the vesicle diameter, *r_pl_* is the protein-to-lipid ratio, *k_cat_* is the turnover number, *M_prot_* is the molecular mass of the protein and *A_lip_* is the area of one lipid molecule in the membrane. Protein-to-lipid mass ratios of 1 : 100 and 1 : 500 are plotted (dashed and continuous lines, respectively) for transporter turnover numbers of 1, 10 and 100 s^−1^ (cyan, orange and black lines respectively). Lines stop at vesicles’ diameters where, on average, one transporter would be present in a vesicle. We assume a single lipid molecular mass to be 800 g mol^−1^ (BNID:101838) with area in a membrane of 0.5 nm^2^ (BNID:106993). Transporter mass was taken as 100 kDa [112]. With our assumptions, *J* = 1.6 × 10^−2^ × π*d*^2^*r_pl_k_cat_*. (**B**) Diffusion of molecules in large vesicles and the scaling of Brownian diffusion time with the diameter of a vesicle. The *y*-axis is given as a mean time needed for a molecule to diffuse from the center of a sphere to its edge. The diffusion coefficients used are based on mobility measurements in the cytoplasm of *E. coli* for NBD-glucose (50 µm^2^ s^−1^; green line, [25]), GFP (10 µm^2^ s^−1^; cyan, [34]), LacZ-GFP homotetramer (0.8 µm^2^ s^−1^; orange, [25]) and free 30S ribosomal subunit (0.12 µm^2^ s^−1^; black, [107]). Dashed line represents GFP diffusion in dilute solution (87 µm^2^ s^−1^ [113]). Diffusion time is given by the equation: *t* = *d*^2^/24*D*; where *d* is the vesicle diameter and *D* is the diffusion coefficient. (**C**) Small vesicles are prone to stochasticity. The number of molecules per vesicle as a function of vesicle diameter is shown. One nanomolar concentration (black line) corresponds to concentrations of a low-abundance protein (∼1 copy per 1 µm^3^ cell), 1 µM corresponds to a medium-high abundant protein (∼10^3^ copies per 1 µm^3^ cell, [114]), 1 mM is representative of the abundance of a metabolite (e.g. amino acid), and 100 mM corresponds to most abundant solutes in cells (K^+^, glutamate) [20]. The number of molecules is given by the equation: *N* = 4/3π(*d*/2)^3^*N*_A_*C*; where *d* is the vesicle diameter, *N_A_* is Avogadro number, *C* is molar concentration. (D) Number of genes in synthetic cells is limited by vesicle size. The volume fraction of the genetic material as a function of vesicle diameter is shown. The black line represents volume fraction taken by the *E. coli* chromosome (4.6 Mbp; BNID:100269), orange line corresponds to the chromosome size of *P. ubique* (1.3 Mbp, [115]), cyan line is associated with the chromosome of JCVI-syn3A [106], and green line represents an approximate size of a 100-gene synthetic chromosome (assuming 1000 bp per gene, [116]). The volume fraction of the genetic material is based on the volume fraction of the *P. ubique* chromosome [115] in 0.01 µm^3^ volume [63]. The volume fraction is given by the equation: Φ = (3*N*_bp_*V*_bp_)/(4π(*d*/2)^3^); where *d* is the vesicle diameter, *N_bp_* is the number of base pairs and *V_bp_* is the volume of a single base pair (here 2.2 nm^3^).

The size and shape of the vesicles determine (i) whether the membrane transport will be a limiting process; (ii) whether molecules will have to diffuse over long or short distances; and (iii) whether stochasticity will play a role or not. The turnover number of transporters varies from 0.001 to 1000 s^−1^ with micronutrient (vitamin) importers typically being the slowest, whereas the Na^+^/H^+^ antiporter NhaA is one of the fastest with a turnover of 1000 s^−1^. Assuming a vesicle with a diameter of 0.2 µm and 20 transporters with a turnover number of 10 s^−1^, the maximum flux of a given solute into the vesicles would be 3.3 × 10^−22^ mol s^−1^. In a vesicle of 20 µm diameter, the flux through the membrane would be 10^4^ times bigger but the volume would be 10^6^ times larger (Figure 3A). With an increase in volume, spherical synthetic cells will inevitably become transport limited. Increasing the protein-to-lipid ratio helps to a certain point, but soon the membrane becomes saturated with proteins. One of the ways living cells deal with changing the surface-to-volume scaling is to alter their shape. Many cells become filamentous when faced with starvation, while some halophiles (organisms living in high salt) are flat disks [86,87] (Figure 2). In synthetic cells, a change in shape can be obtained by, for instance, osmotic shift [88,89], by forming tethered vesicle networks [90], by the use of microfluidic [91] or electric fields [92]. Shape also determines the maximum distance that transported molecules would have to diffuse from the membrane. In the case of a spherical vesicle, its radius gives the longest distance from the membrane. In lipid nanotube structures, which can be formed between two vesicles, the maximum distance a newly transported molecule will have to diffuse is in the range of 50–150 nm (average radius of lipid nanotubes) [93]. In Brownian diffusion, which is driven by thermal fluctuations, the traveled distance scales linearly with the square root of time, as given by the Stokes–Einstein equation [94]. This makes diffusion slow over long distances (>10 s for 20 µm vesicle, see Figure 3B) [94]. In these cases, filamentous structures or vesicular systems in which the internal medium is confined to regions near the membrane, like the cytoplasm in giant bacteria [86], would have an advantage over spherical vesicles, since the surface-to-volume ratio is higher. Nanotubes are not widely used in synthetic biochemistry because of their high surface free energy and a forced membrane curvature. This results in high instability of the structure, which tend to spontaneously form a sphere to lower the surface free energy [95]. Studies have shown how nanotubes connected to spherical vesicles composed of phospholipids allow the diffusion of molecules from one vesicle to another [96–98].

**Figure 4.**
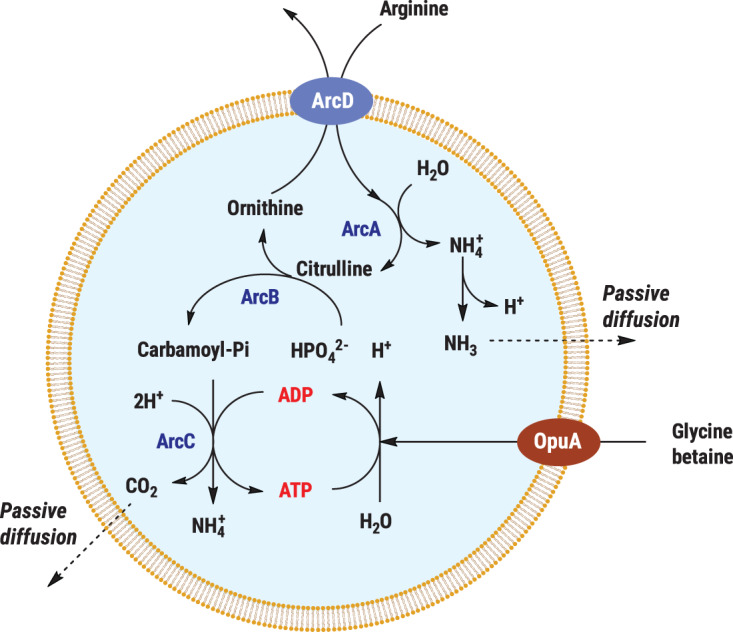
Schematic representation of the arginine breakdown pathway fueling ATP production inside vesicles.

### Stochasticity

Another parameter to consider in the construction of synthetic cells is stochasticity. Stochastic effects are random events that depend on the size and the shape of the (vesicle) system, and on the concentration of its components, e.g. the enzymes enclosed in the vesicle lumen and the proteins embedded in the membrane. In small volumes such as sub-micrometer vesicles, macromolecules will be present in low numbers due to their large size (Figure 3C), which results in higher stochasticity. Stochastic events are not unique to synthetic systems but are also common in living cells [99].

### Encapsulation and reconstitution efficiency

Encapsulation and reconstitution are semi-quantitative stochastic processes. The starting concentration of each component is known, but molecules do not always distribute equally over the vesicles [100]. Interestingly, macromolecules can ‘superfill’ vesicles as was shown for ferritin in small liposomes, and here the smaller the vesicle, the higher the level of ferritin accumulation [100]. In some cases, the encapsulation is affected by the electrostatic attraction or repulsion of the target molecules from the membrane [100]. PURE is a minimal defined transcription and translation system, composed of some 80 molecular components that all have to be present in sufficient amounts for protein synthesis [101]. The probability to encapsulate the complete PURE system in vesicles of 0.1 µm diameter is extremely low (10^−26^), yet protein production has been observed in ensembles of such vesicles. The authors conclude that the production of GFP comes from the superfilled vesicles, since it would be impossible to obtain vesicles with all necessary components if the encapsulation would be random [101]. Less than one in a hundred vesicles was estimated to be superfilled, resulting in a heterogenous population with sparse active systems [100]. PURE encapsulated in 4 μm diameter vesicles produces the fluorescent protein in ∼30% of vesicles [102]. For constructing synthetic cells, it can be advantageous to have a large encapsulation volume to ensure that all the necessary components are present in a large fraction of the vesicles, yet their concentration will be much lower than in the superfilled small vesicles. Minimization of the number of components is important to minimize stochasticity (Figure 3B). As for encapsulation, reconstitution of membrane proteins faces similar problems — poor yields and heterogeneity. A small potassium channel KvAP has been successfully reconstituted in small liposomes up to 1 : 10 protein-to-lipid mass ratio and in giant liposomes up to 1 : 20 [103,104]. Both of those numbers fall short compared with the 3 : 2 mass ratio found in bacteria like *E. coli* [105].

### Volume of genetic information

In small synthetic cells, the genetic material can occupy a significant volume fraction. A genome of ∼500 genes has been demonstrated as the minimal genome needed to form an autonomously propagating cell [106]. A single copy of it would occupy ∼10% of the volume of a 250 nm diameter vesicle. The DNA volume fraction of *E. coli* is ∼1%; for *P. ubique*, one of the smallest known bacteria, the DNA volume fraction is 30% (Figure 3D). Additionally, in *E. coli*, the chromosome excludes large complexes, like the polysomes (mRNA translated by multiple ribosomes), the effect of which would be even more pronounced in smaller (synthetic) cells [107]. Therefore, in synthetic cells, the genetic material could significantly limit the reaction volume of the translational machinery.

### Extremophilic components

Efforts in synthetic biology tend to center around mesophilic conditions and components, that is, those of most commonly used laboratory cells. Extremophiles, organisms living in harsh environments, evolved ways to deal with the unwelcome conditions [8,39,108–110]. Depending on the desired application of the synthetic system, (macro)molecules derived from relevant extremophiles could have advantages such as more stable enzymes or membranes. We know now that the conditions the early life forms experienced were most likely far more extreme in terms of temperature, pressure, radiation and concentration of ions than those of the majority of today's cells [111].

## Conclusions

Cells have evolved mechanisms to deal with physicochemical homeostasis. Bottom-up built synthetic cells also require such control, and in this paper, we described the relevant physicochemical conditions that should be taken into account when designing synthetic cells. To obtain optimal reactivity of the system, pH, ionic strength, excluded volume and surface-to-volume ratio need to be set and maintained at a certain level. Some of the physicochemical parameters will change as the system is active and thus homeostatic mechanisms need to be implemented. Major questions the field needs to address are (i) which physicochemical parameters require control and (ii) what is the simplest way to achieve the control.

## Summary

- Physicochemical conditions affect the activity of biological systems, both individually and synergistically.
- Physicochemical homeostasis is key for living and synthetic cells.
- ATP synthesis and generation of electrochemical ion gradients are needed to drive endergonic reactions and are at the heart of physicochemical homeostasis.
- Depending on the purpose, the size and the shape of synthetic cells, the impact of the physicochemical conditions will differ.
- Synthetic biologists need to find minimal systems to control the physicochemical homeostasis in manmade cells.

## Abbreviations

ADP: adenosine diphosphate
ATP: adenosine triphosphate
BNID: Bionumbers ID
DNA: deoxyribonucleic acid
GFP: green fluorescent protein
GUVs: giant unilamellar vesicles
LUVs: large unilamellar vesicles
mRNA: messenger RNA
PC: phostidylcholine
PE: phosphatidylethanolamine
PG: phosphatidylglycerol
Pi: phosphate
PIP: phostidylinositol phosphate
PS: phostidylserine
PURE: protein synthesis using recombinant elements
RNA: ribonucleic acid
SUVs: small unilamellar vesicles
